# Investigation of Chemomarkers of Astragali Radix of Different Ages and Geographical Origin by NMR Profiling

**DOI:** 10.3390/molecules20023389

**Published:** 2015-02-17

**Authors:** Lu Zheng, Mei Wang, Emmanuel Ibarra-Estrada, Changsheng Wu, Erica Georgina Wilson, Robert Verpoorte, Petrus Gerardus Leonardus Klinkhamer, Young Hae Choi

**Affiliations:** 1 Shanghai Haini Pharmaceutical Co. Ltd., Yangtze River Pharmaceutical Group, Shanghai 201318, China; E-Mail: lucyzheng17@163.com; 2 Natural Products Laboratory, Institute of Biology, Leiden University, Sylviusweg 72, Leiden 2333 BE, The Netherlands; E-Mails: mei.wang@tno.nl (M.W.); emmanuel.ibarra8@gmail.com (E.I.-E); c.wu.3@umail.leidenuniv.nl (C.W.); e.g.wilson@biology.leidenuniv.nl (E.G.W.); verpoort@chem.leidenuniv.nl (R.V.); 3 Sino-Dutch Centre for preventive and Personalized Medicine, Zernikedreef 9, Leiden 2333 CK, The Netherlands; 4 Plant Ecology and Phytochemistry, Institute of Biology, Leiden University, Sylviusweg 72, Leiden 2333 BE, The Netherlands; E-Mail: p.g.l.klinkhamer@biology.leidenuniv.nl

**Keywords:** *Astragalus membranaceus*, metabolomics, nuclear magnetic resonance spectroscopy, saponins, isoflavonoids

## Abstract

*Astragalus* roots from *Astragalus membranaceus* Bunge or *Astragalus membranaceus* var. mongholicus (Bunge) Hsiao are among the most popular traditional medicinal plants due to their diverse therapeutic uses based on their tonic, antinephritic, immunostimulant, hepatoprotectant, diuretic, antidiabetic, analgesic, expectorant and sedative properties. Currently, the herb is produced or cultivated in various sites, including 10 different locations in China with very diverse environmental conditions. These differences affect their metabolic pools and consequently their medicinal properties. The comparative metabolic profiling of plants of different geographical origins or ages could contribute to detect biomarkers for their quality control and thus guarantee the efficacy of the herbal medicines produced with this drug. In this paper nuclear magnetic resonance spectroscopy (NMR)-based metabolomics was applied for to plants of different origins and age for this purpose. The results of this study show that in the set of samples evaluated, age is more discriminating than geographical location. The quantity of individual flavonoids and some primary metabolites contributed most to this age differentiation. On the other hand, based on the analysis of orthogonal partial least square (OPLS) modeling, the marker metabolites for the geographical origin were saponins and isoflavonoids.

## 1. Introduction

The herbal medicine Radix Astragali is most commonly prepared from the dried root of *Astragalus membranaceus* Bunge or *A. membranaceus* var.* mongholicus* (Bunge) Hsiao (Leguminosae). It is one of the most well-known traditional medicines in far-East Asia having been used in China for about 2000 years. Due to its widespread consumption for a large number of disorders, it is officially listed in the Pharmacopeias of China, Japan and Korea. It is used mainly as a tonic and for its hepatoprotective, diuretic, analgesic, expectorant and sedative properties. It has also been employed for the treatment of diabetes and nephritis [1,2,3,4].

In view of all these bioactivities, the demand for *Astragalus* roots in the world market and particularly in China and Korea has been steadily rising. Botanically, the genus *Astragalus* comprises 278 species, two subspecies, 35 varieties and two forma in China, of which only 12 can be used as medicinal plants [5]. Officially, the Chinese Pharmacopoeia defines Radix Astragali as the radices of *Astragalus membranaceus* Bunge and *A. membranaceus* Bunge var. *mongholicus* (Bunge) Hsiao [6,7] coinciding with other monographs [8]. This is reasonable considering that *A. membranaceus* and *A. membranaceus* var*. mongholicus* have a similar chemical composition and genomic DNA sequences. In spite of this, it is possible to find eight more species of *Astragalus* and a substitute, *Hedysarum polybotrys* Handel-Mazzetti, especially in northern China, all of which are commercially available under the name of Radix Astragali, but are, in fact, adulterants [9]. The constituents most often associated with the activity of Radix Astragali are isoflavonoids, saponins, polysaccharides, *γ*-aminobutyric acid (GABA), and various trace elements [2,8].

Nowadays, Radix Astragali is mostly prepared from cultivated plants [10]. Both *A. membranaceus* and *A. membranaceus* var*. mongholicus* are cultivated mainly in the northern (provinces of Shanxi, Neimenggu, and Hebei) and the northeastern region (Heilongjiang) of China [10]. Recent studies also indicate that the best quality of Radix Astragali is produced in Shanxi [10]*.* For example, the *A. membranaceus* from Shanxi has been reported to contain more health-promoting trace elements than Heilongjiang- and Neimengu- grown material that contains more lead [11]. It is thus important to be able to distinguish the origin of the plant material to guarantee its efficacy and safety as a herbal medicine.

**Figure 1:**
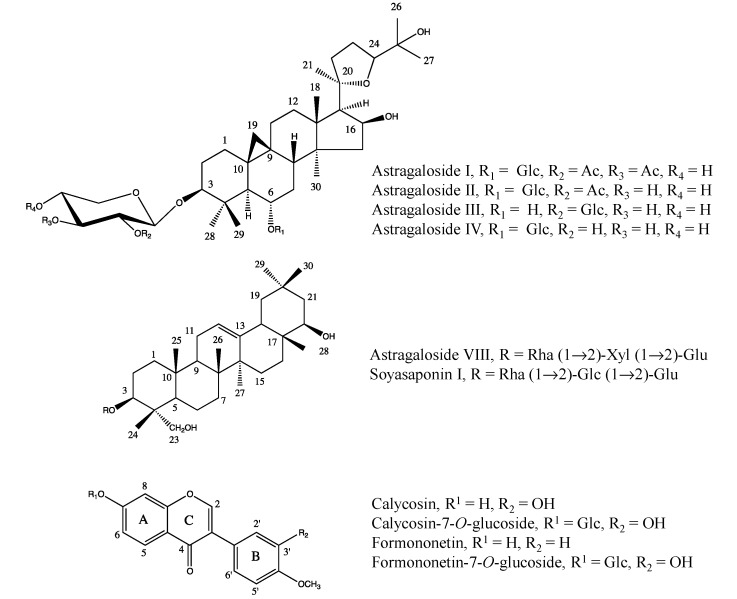
Chemical structures of typical saponins and flavonoids of *Astragalus* roots.

Both primary and secondary metabolites have been identified as biologically active constituents of *Astragalus* roots. The bioactive primary metabolites are mainly polysaccharides. Fang* et al.* [12] isolated three polysaccharides, astragalan I, II and III with a molecular mass ranging from 12,300 to 36,300 Da from the aqueous extract of the roots of *A. membranaceus* var.* mongholicus*; of these, astragalan II and III were found to be composed mainly of D-glucose while astragalin also contained D-galactose, L-arabinose and traces of pentose. Another four homogenous polysaccharides have been identified: two glucans, AG-1 and AG-2 [13] and two heterosaccharides (AH-1 and HA-2). In general, these polysaccharides have been associated to a wide spectrum of effects on the immunological system [14,15,16,17]. Saponins and flavonoids, particularly isoflavonoids, as shown in Figure 1, have been found to be the most characteristic secondary metabolites of *A. membranaceus* [2,8,18].

In the case of saponins, Kitagawa* et al.* [19] reported the presence of triterpene oligoglycosides in the roots of Korean *A. membranaceus* with two aglycones: cycloastragenol, a 9,19-cycloanostane type triterpene, or astragenol, a lanost-9(11)-ene triterpene which is formed secondarily as an artefact from cycloastragenol. Astragenol is the aglycone common to 10 of the 11 saponins known as astragalosides. In addition to cycloastragenol or astragenol-type saponins, astragaloside VII and soyaponin I, two minor oleanane-type triterpenic saponins have also been isolated [20]. Saponins have been associated with the hepatoprotective effects observed for *Astragalus* root preparations [21].

The main isoflavonoids of *A. membranace*us roots are calycosin, formononetin and 3-hydroxy-9,10-dimethoxypterocarpan. In general these and other isoflavones have been related to the antioxidant effects observed in *Astragalus* extracts. Formononetin, calycosin and their glycosides [18] have been found to inhibit glutamate-induced cell injury, generally through the increase of the antioxidant enzymes, superoxide dismutase (SOD) and glutathione peroxidase (GSH Px) and the inhibition of the release of lactate dehydrogenase (LDH). These isoflavones from *Astragalus* roots have also been proved to inhibit lipid peroxidation by reactive oxygen species [22].

Among the metabolites of *Astragalus* roots, a saponin, astragaloside IV, is normally used as a quality control marker [10]. However, the level of this and other typical constituents varies according to their origin, as has been shown by thin layer chromatography (TLC), high-performance liquid chromatography (HPLC), and a colorimetric method [8,9,10,23]. In recent years, diverse analytical platforms such as nuclear magnetic resonance spectroscopy or mass spectrometry have been applied to medicinal plants as an unbiased approach to the search for chemo- or biomarkers both for chemotaxonomical purposes and/or quality control. Among them, a number of analytical methods have been developed to attempt to identify the origin of commercial Radix Astragali.

In one of these studies, ICP-MS and ^1^H-NMR were applied to discriminate the geographical origin of *A. membranaceus* samples obtained from Korea and China. Using four different classification methods, several amino- or organic acids were found to be potential markers for the differentiation of the origin [24]. For another study, the same researchers also used ^1^H-NMR and UPLC-MS to study the effect of the post-harvest peeling procedure on the chemical composition of the roots, finding that peeled roots exhibited significant changes in their metabolomic profile, showing the loss of some primary metabolites and the increase of xylem-related compounds and formate, which is produced in response to wound stress [25]. However, despite these and other extensive metabolic profiling studies of *Astragalus* roots, no activity-related markers that could help to distinguish samples on the basis of their geographical origin as an indicator for their quality control had yet been detected. It was thus particularly important to detect any differences concerning levels of the secondary metabolites such as saponins and isoflavonoids that had been associated to the clinical efficacy of this medicinal drug. Among the possible analytical platforms that can be used for a metabolomic study, NMR has several advantages such as its high-speed, ease of quantitation and compound identification, robustness of obtained data and reproducibility. In addition, an NMR spectrum can be considered to be a fingerprint of a plant, which can be used over a long period of time. However, so far most of the applications of NMR-based metabolomics to *Astragalus* roots had been limited to primary metabolites.

In this study, *Astragalus* roots collected from diverse locations in China, e.g., Jilin, Heilongjiang, Neimenggu, Hebei, Shanxi, Xinjiang and Shandong provinces in China were analyzed by ^1^H-NMR-based metabolomics supported by two-dimensional NMR. From the results, possible chemomarkers for the location of the plants are proposed.

## 2. Results and Discussion

### 2.1. ^1^H-NMR Characterization of Saponins and Isoflavonoids of Astragalus Roots

Metabolomics has been applied recently to discriminate *Astragalus* roots collected from diverse places in Korea and three provinces of China: Shandong, Hebei, and Liaoning [24]. In that study, some discriminating primary metabolites were identified, including organic acids (acetic acid, fromic acid, fumaric acid, malic acid) and amino acids (alanine, arginine, proline, valine) as well as sucrose and a few inorganic substances. However there was scarce information on the variation of secondary metabolites between samples from different locations.

The two major activity-related secondary metabolite groups in *Astragalus* are saponins and isoflavonoids, though their content is very variable. Thus, it was reasonable to begin the search for chemomarkers of this drug with these groups. ^1^H-NMR has many advantages as a tool for metabolic profiling but when used directly—without any hyphenation to a chromatographic system—the signals in the obtained spectrum will mostly likely show a great deal of overlapping. Therefore, the first step of ^1^H-NMR-based metabolomics is to pick out the characteristic signals of possible metabolites present in the samples among the many other signals.

Saponins contain two kinds of moieties, a hydrophobic steroidal or triterpenic aglycone and a hydrophilic part made up of sugars. In the ^1^H-NMR structural elucidation of saponins, the most characteristic ^1^H resonances are from anomeric and methyl signals. Depending on the sugars and their bonding, each anomeric proton has its particular chemical shift and coupling constants. However, these anomeric protons are detected in the crowded crown region, δ 4.5–δ 5.5, where signals of many anomeric protons of free sugars and residual HDO are also detected and thus overlap.

Other important characteristic signals of saponins correspond to methyls detected in the range of δ 0.6–δ 1.5. The methyl signals are mostly singlets with a few highly intense doublets corresponding to 3 protons. In order to investigate the feasibility of the methyl signals as marker ^1^H resonances, H-18, H-21, H-26, H-27, H-28, H-29, and H-30 of astragaloside I, II, III, and IV (Figure 1) were assigned using the 2D-NMR experiments, HSQC and HMBC. Particularly in the HMBC spectrum, each methyl signal can be well distinguished by characteristic correlations: H-18/C-13 and C-17, H-21/C-20 and C-17, H-26 and H-27/C-25 and C-24, H-28 and H-29/C-3, and H-30/C-13. The assignment of the seven methyl group protons and cyclopropane moiety of four astragalosides is listed in Table 1, both in CD_3_OD and CD_3_OD -KH_2_PO_4_ buffer in D_2_O (1:1, v/v).

**Table 1:** ^1^H-NMR chemical shifts (ppm) and coupling constants (Hz) of methyls and H-19 of astragaloside I, II, III, and IV in CD_3_OD ^a^ (Solvent 1) and CD_3_OD-KH_2_PO_4_ buffer in D_2_O (1:1, v/v) ^b^ (Solvent 2).

No. of H	Solvent	Astragaloside I	Astragaloside II	Astragaloside III	Astragaloside IV
H-18	1	1.24 (s)	1.24 (s)	1.25 (s)	1.25 (s)
	2	1.24 (s)	1.24 (s)	1.25 (s)	1.24 (s)
H-19a	1	0.26 (d, 4.4 Hz)	0.26 (d, 4.5 Hz)	0.38 (d, 4.3 Hz)	0.26 (d, 4.4 Hz)
	2	0.35 (d, 4.4 Hz)	0.35 (d, 4.5 Hz)	0.40 (d, 4.3 Hz)	0.37 (d, 4.4 Hz)
H-19b	1	0.58 (d, 4.4 Hz)	0.58(d, 4.5 Hz)	0.55 (d, 4.3 Hz)	0.59 (d, 4.4 Hz)
	2	0.57(d, 4.4 Hz)	0.57(d, 4.5 Hz)	0.54 (d, 4.3 Hz)	0.59 (d, 4.4 Hz)
H-21	1	1.19 (s)	1.18 (s)	1.23 (s)	1.22 (s)
	2	1.20 (s)	1.22 (s)	1.23 (s)	1.22 (s)
H-26	1	1.12 (s)	1.12 (s)	1.12 (s)	1.13 (s)
	2	1.26 (s)	1.26 (s)	1.14 (s)	1.27 (s)
H-27	1	1.22 (s)	1.22 (s)	1.25 (s)	1.25 (s)
	2	1.26 (s)	1.26 (s)	1.29 (s)	1.29 (s)
H-28	1	1.29 (s)	1.29 (s)	1.32 (s)	1.28 (s)
	2	1.27 (s)	1.27 (s)	1.28 (s)	1.28 (s)
H-29	1	0.90 (s)	0.90 (s)	1.02 (s)	1.01 (s)
	2	0.90 (s)	0.90 (s)	1.01 (s)	0.99 (s)
H-30	1	1.01 (s)	1.00 (s)	0.99 (s)	1.02 (s)
	2	0.99 (s)	0.99 (s)	0.99 (s)	1.01 (s)

Notes: ^a^ Chmical shifts were calibrated to TMS (0.44 mM) at 0.0 ppm; ^b^ Chemical shifts were calibrated to TMSP (0.29 mM) at 0.0 ppm.

All the major *Astragalus* saponins share a common aglycone, cycloastragenol, which is substituted with different sugar moieties at the C-3 or C-6 positions. This chemical resemblance makes it very difficult to distinguish the astragalosides. Furthermore, these methyl signals from saponins might also overlap with other methyl signals from amino acids such as isoleucine, leucine, and valine, which are commonly detected as free amino acids in plant extracts. However, cycloastragenol has a characteristic cyclopropane moiety in the ring of C-9/C-10/C-19 that is absent in most common triterpenoids such as oleanane- or ursane-type triterpenoids. The two protons of H-19 are detected as doublets upfield-shifted to a non-crowded region, one of them to δ 0.2–δ 0.4 and the other to δ 0.5–δ 0.6 (Table 1). The absence of glycosylation at C-6 produces a downfield shift of the H-19 protons of δ 0.38 (d, *J* = 4.3 Hz) in the case of astragaloside III as compared with d 0.26 (d, *J* = 4.4 Hz) of other astragalosides in the solvent of CD_3_OD-KH_2_PO_4_ buffer in D_2_O (1:1, v/v). These signals that correspond to the cyclopropane moiety can be used as target signals for the saponins in the ^1^H-NMR spectrum of *Astragalus* roots

**Figure 2:**
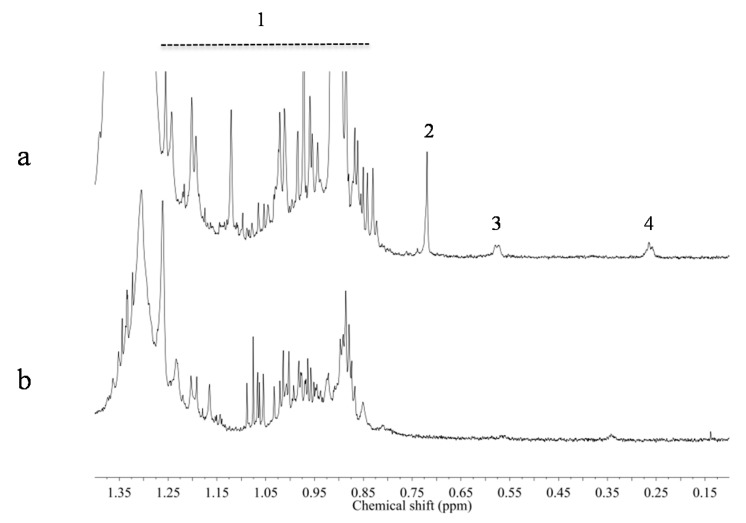
^1^H-NMR spectra of *Astragalus membranaceus* roots collected in Shanxi province in China (No. of voucher specimen: 20131206-2). (**a**) In CD_3_OD; (**b**) CD_3_OD-KH_2_PO_4_ buffer in D_2_O (1:1, v/v), 1: methyl signals of astragaloside I, II, III, IV, isoleucine, leucine, and valine, 2: H-24 of astragaloside VIII and soyasaponin I, 3: H-19a of astragalosides, 4: H-19b of astragalosides.

The intensity of signals and consequently the sensitivity of the ^1^H-NMR method is generally affected by the solvent. To choose the best solvent, the spectra of CD_3_OD or CD_3_OD-KH_2_PO_4_ buffer in D_2_O (1:1, v/v) extracts of *Astragalus* were compared to determine which showed the highest resolution or intensity of the methyl and H-19 signals (Figure 2). Most methyl signals such as H-18, H-21, H-26, H-27, H-28, H-29, and H-30 overlapped with other signals, but the H-19 signals were clearly detected in a non-crowded region in both cases. However, the highest sensitivity for these signals was observed in extracts of the non-aqueous solvent, CD_3_OD. This is probably due to the fact that saponins generate micelles in an aqueous solvent resulting in signal broadening. Thus, in this case, CD_3_OD proved to be a better solvent than CD_3_OD-KH_2_PO_4_ buffer in D_2_O (1:1, v/v), which is commonly used for NMR-based plant metabolomics.

The advantage of using CD_3_OD rather than aqueous solvents was not limited to the detection of the H-19 of cycloastragenol saponins. The H-23 signal of another type of saponin—soyasapgenol B, an oleanane-type triterpenoid (Figure 1)—was also clearly detected with higher intensity in the non-aqueous solvent.

Apart from saponins, *Astragalus* roots also contain a high amount of isoflavonoids. To investigate characteristic isoflavonoid ^1^H-NMR resonances, the spectra of four reference compounds—calycosin, formononetin and their 7-*O*-glucosides—in CD_3_OD and CD_3_OD-KH_2_PO_4_ buffer in D_2_O (1:1, v/v) were recorded. In the Figure 2, singlets corresponding to H-2 resonances in the C-ring can be observed to be well distinguished from other signals in the downfield region around δ 8.1–δ 8.4; this is due to the α-effect of oxygen and the effect of the β-position from the ketone group. If a B-ring is attached to H-2 as in the case of common flavones such as apigenin or luteolin, the H-3 of the C-ring proton is detected around δ 6.9 as a singlet. Thus, the greatly downfield-shifted H-resonance of H-2 can be used as an isoflavonoid signal in ^1^H-NMR-based metabolomics.

**Figure 3:**
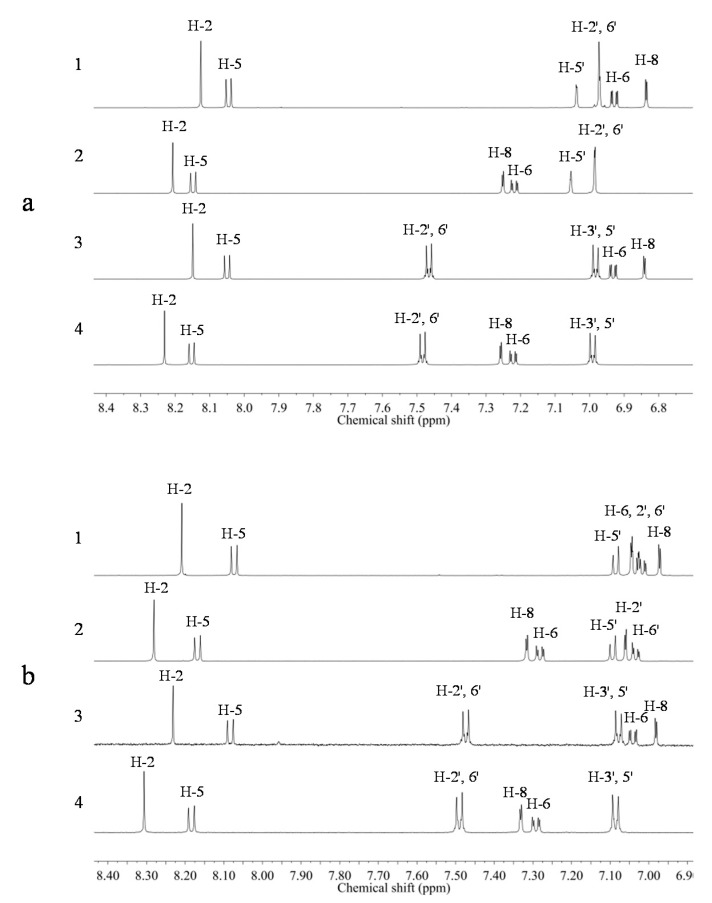
^1^H-NMR spectra of calycosin (1), calycosin-7-*O*-glucoside (2), formononetin (3), and formononetin-*O*-glucoside (ononin, 4). (**a**) In CD_3_OD; (**b**) CD_3_OD-KH_2_PO_4_ buffer in D_2_O (1:1, v/v).

In addition to H-2, calycosin and formononetin have a hydroxyl group at the C-7 position, which causes a downfield shift of the H-5 proton signal to δ 8.2 (d, *J* = 8.8 Hz) (Figure 3). Calycosin and formononetin can be differentiated by the pattern of their B-ring. Formononetin has a typical 1,4-disubstituted benzene ring pattern, two multiplets at *ca.* δ 7.0 (H-3' and H-5') and *ca.* δ 7.5 (H-2' and H-6').

Additionally, the presence of glucose can be detected by the shift of H-6 and H-8 protons. The *O*-glycosidic linkage at the C-7 position causes a downfield shift of the signals of both protons of *ca.* 0.3 ppm as compared with that of their corresponding aglycones.

**Figure 4:**
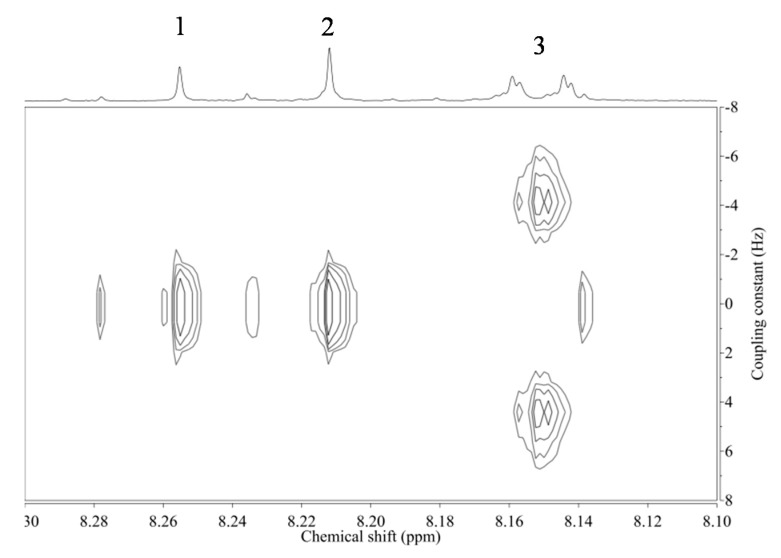
^1^H-^1^H-J-resolved spectrum of *Astragalus membranaceus* roots collected in in the Shanxi province in China (No. of voucher specimen: 20131206-5) in CD_3_OD. 1: H-2 of formononetin-7-*O*-glucoside, 2: H-2 of calycosin-7-*O*-glucoside, 3: H-5 of formononetin-7-*O*-glucoside and calycosin-7-*O*-glucoside.

The presence of a low amount of isoflavonoids was confirmed in the CD_3_OD extract of *Astragalus* roots. Two kinds of H-2 and H-5 were clearly detected in the ^1^H-NMR spectrum and the coupling constants were confirmed by ^1^H-^1^H-J-resolved spectrum (Figure 4). Based on the information of the chemical shifts of the reference compounds, the two major isoflavonoids were assigned to be calycosin-7-*O*-glucoside and formononetin-7-*O*-glucoside (ononin).

In addition to saponins and flavonoids, the ^1^H-NMR spectrum of *Astragalus* roots revealed the presence of a number of primary metabolites, including amino acids (alanine, arginine, asparagine, leucine, isoleucine, proline, and valine), organic acids (citric-, formic and malic acid), and sugars (sucrose and glucose) as well as γ-aminobutyric acid (GABA) and choline. Among the metabolites detected, sucrose was found to be the most abundant, accounting for over 15% of the dry weight of *Astragalus* roots.

### 2.2. Multivariate Data Analysis of ^1^H-NMR Spectra to Investigate the Geographical Variation of Astragalus Roots

The ^1^H-NMR data were further reduced by principal component analysis to investigate the grouping between the samples. Apart from obvious factors such as species and variety, there are at least two other factors that can potentially influence the metabolite profile of *Astragalus* root samples: age and geographical origin. The samples investigated in this study (Table 2) were identified as *Astragalus membranaceus* var. mongholicus, *Asgragalus membranaceus*, and *Hedysarum polybotrys*. *Astragalus membranaceus* var. mongholicus and *A. membranaceus* are defined as Radix Astragali but *Hedysarum polybotrys* is used as a red-coloured substitute [9].

**Table 2:** Sample information of *Astragalus* roots evaluated in this study.

Sample Code *	Voucher Specimen	Collection	Wild/Cultivated	Age (Years)	Source of Materials
1	304032-1302004	^a^	Cultivated	2–3	^g^
2	20131018	^a^	Cultivated	2–3	^h^
3	201210	^a^	Cultivated	2–3	^i^
4	304032-1306009	^a^	Cultivated	2–3	^i^
5	20131222-2	^a^	Cultivated	2–3	^j^
6	121103	^b^	Cultivated	2–3	^k^
7	20131120-1	^b^	Cultivated	2–3	^l^
8	20131120-2	^b^	Cultivated	2–3	^l^
9	20131122-3	^b^	Cultivated	2–3	^j^
10	20131119	^b^	Cultivated	2–3	^h^
11	21031020	^b^	Cultivated	2–3	^h^
12	20131206-1	^c^	Wild	5	^m^
13	20131206-2	^c^	Cultivated	2	^m^
14	20131206-3	^c^	Wild	20	^m^
15	20131206-4	^c^	Wild	12	^m^
16	20131206-5	^c^	Wild	30	^m^
17	20131222-1	^c^	Cultivated	2–3	^j^
18	20131203	^d^	Wild	10	^n^
19	20131207-1	^d^	Wild	10	^n^
20	20131207-2	^d^	Wild	5	^n^
21	20131209-1	^d^	Wild	10	^n^
22	20131209-2	^d^	Cultivated	2–3	^n^
23	20131210	^d^	Wild	10	^n^
24	DA13121001	^e^	Cultivated	2–3	^o^
25	DA13121002	^e^	Cultivated	2–3	^o^
26	DA13121003	^e^	Cultivated	2–3	^o^
27	DB13121001	^e^	Cultivated	2–3	^p^
28	DB13121002	^e^	Cultivated	2–3	^p^
29	DB13121003	^e^	Cultivated	2–3	^p^
30	20131222-4	^f^	Cultivated	2–3	^j^

Notes: * Sample code (1–17 and 27–30) identified as *Astragalus membranaceus* var. mongholicus (Bunge); Sample code (18–23) identified as *Astragalus membranaceus* Bunge; Sample code (24–26) identified as *Hedysarum polybotrys* Hand-Mazz; ^a^ Neimenggu province, China; ^b^ Gansu province, China; ^c^ Shanxi province, China; ^d^ Heilongjiang province, China; ^e^ Shaanxi province, China; ^f^ Hebei province, China; ^g^ Chengdu Lotus Pond Pharmaceutical Co. Ltd. (Chengdu, China); ^h^ Anhui Bozhou Medicine Market; ^i^ Anguo City Gingeng Antler Herbs Ltd. (anguo, China); ^j^ Anguo Medicine Market; ^k^ Anguo City Golden Grass Chinese Herbal Medicine Co. Ltd. (Anguo, China); ^l^ Bozhou City Wikang Herbs Stack; ^m^ Huiyuanwansheng Astragalus Development Co. Ltd.; ^n^ Heilongjiang Daxinganling Wild Collected; ^o^ Shaanxi Quinling Mountains; ^p^ Cultivated at the junction of Gansu and Shaanxi.

**Figure 5:**
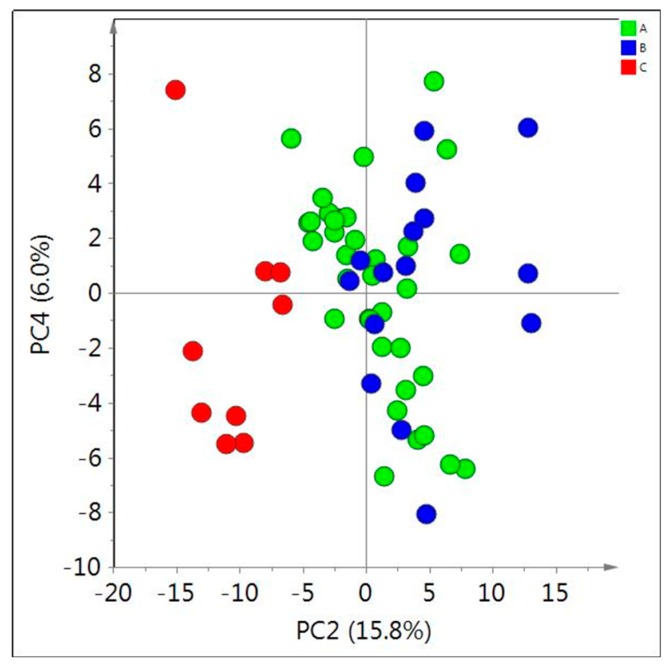
Score plot of principal component analysis (PCA, PC2/PC4) obtained from ^1^H-NMR data of in CD_3_OD extracts of 2–3 year old *Astragalus membranaceus* var. mongholicus (A); *Asgragalus membranaceus* (B); and *Hedysarum polybotrys* (C).

Prior to the investigation of the effect of age and location, the metabolic difference between the species and varieties were evaluated by PCA of their ^1^H-NMR data. For this, samples of a similar age (2–3 years old) were tested. As shown in Figure 5, no clear separation was observed between *A. membranaceus* var. mongholicus and *A. membranaceus*. However, *H. polybotrys* was distinguished from others by the low amount of isoflavonoids and saponins, which is consistent with the previous data [9].

Geographical location can affect the metabolome of a plant due to a number of external factors comprising temperature, soil conditions, altitude rain and drought, whereas the effect of age is caused by internal factors related to gene expression during the plant development. Because of this wide range of variables, a large number of samples would be needed to reach significant conclusions. This problem can be circumvented by assuming that there is a specific marker for each developmental stage of the plant as well as specific markers for the sites of the collection. Markers which will be sufficiently selective as to stand out above the variability can be detected by means of supervised multivariate data analysis, in which specifically the differences between some classes are analysed, such as, for example, the difference between samples of different age. With this approach, all random variability such as that due to diurnal and seasonal changes for example, will be appear only as noise, so that, if present, specific markers will be revealed. Thus, after PCA, we applied partial least square (PLS) and orthogonal partial least square (OPLS) modeling, two supervised multivariate data analysis methods.

**Figure 6:**
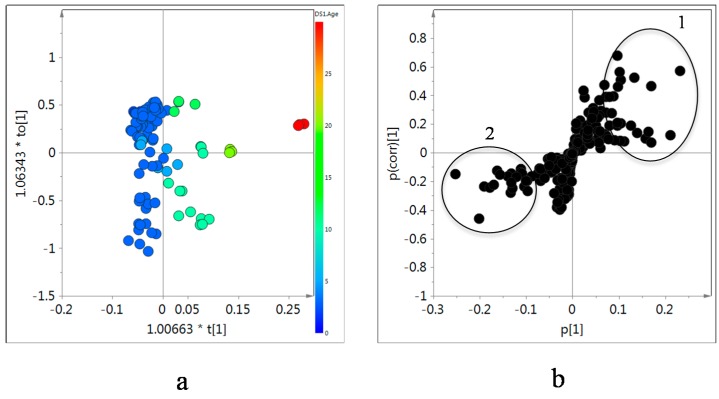
Score (**a**) and S-plot (**b**) of orthogonal partial least square (OPLS) modeling obtained from ^1^H-NMR data of in CD_3_OD extracts of *Astragalus* roots as X-data and age of each sample as Y-data. 1: H-2, H-5, H-6 and H-8 of isoflavonoids, H-1 and H-2' of sucrose, and H-3 of alanine, 2: H-2 and H-3 of asparagine and aspartic acid, CH_3_ of choline, H-4 of GABA, and H-4 of proline.

As expected, no grouping for age or geographical location of the samples was obtained by PCA since the random variability was too high. The samples were collected from diverse locations and with a wide range of ages. However, OPLS modeling using age as a Y data set showed a clear trend in the OPLS component 1, revealing which metabolites are influenced by ages (Figure 6a). The samples were clearly separated into four groups depending on the age of the roots: 2–9, 9–16, 16–23, and 30 years. Obviously, the orthogonal component in y-axis reflects the large variability but this did not interfere with the age-dependant markers.

The metabolites contributing to the age separation were analyzed by an S-plot of the OPLS modeling (Figure 6b). Older samples showed higher levels of isoflavonoids than younger ones. The signals of H-5, H-6, and H-8 of isoflavonoids including calycosin and formononetin, their 7-*O*-glucosides were found be signals that most contributed differentiate the old *Astragalus* samples. In addition, sucrose and alanine were found to be higher in old samples. In the case of saponins, we could find no difference in the intensity of H-19, the characteristic ^1^H resonances of astragalosides, nor for any other methyl signal throughout all samples.

**Figure 7:**
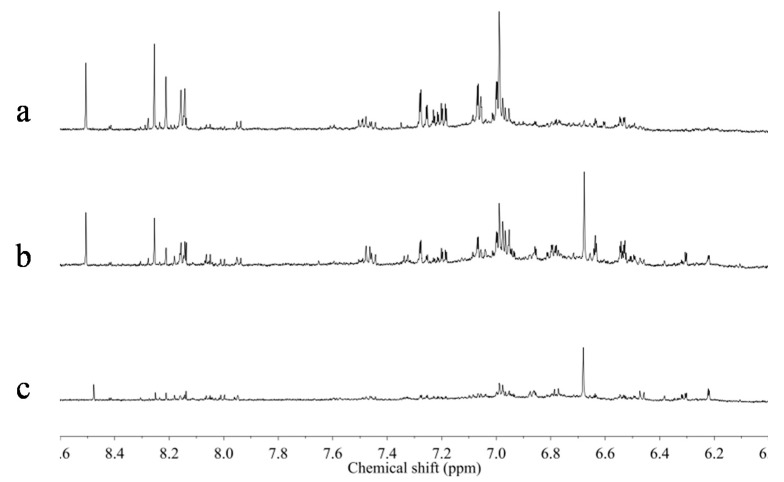
^1^H-NMR spectra (CD_3_OD) of 30 (a), 20 (b), and 5 years (c) old *Astragalus* roots collected from Shanxi province in China.

In the younger roots, higher levels of some primary metabolites including asparagine, aspartic acid, GABA and choline were observed. To confirm this result, ^1^H-NMR spectra of one set of materials, which was collected in the same location but were of different ages, were compared. In Figure 7, the aromatic region of the ^1^H-NMR spectra of 5, 20 and 30, year-old *Astragalus* roots collected from Shanxi province in China were compared. The resonances of isoflavonoids were clearly higher in the older samples than younger ones. However, the effect of age on the metabolome should be further studied on samples of diverse regions and ages because there were only a small number of old-aged samples among all those tested.

A previous study by Ma and colleagues [9] had revealed metabolic differences in *Astragalus* roots during their first 4 years. Total polysaccharides and GABA were reported to be significantly higher in 3-year-old plants, while 1-year-old plants contained the highest amount of total amino acids, which is in accordance with our current data. However, they did not find any significant change in the total isoflavonoid and saponin levels in plants that were up to 4 years old with the exception of astragaloside I that was slightly increased. The different time scale used in the studies could account for the difference in results. As can be seen in Figure 5, the level of isoflavonoids increased only in roots that were more than 10 years old. This change would not thus be detected in the 1- to 4-year-old roots that were evaluated by Ma and colleagues [9]

Neither PCA nor supervised multivariate data analysis (PLS or OPLS-modeling) revealed changes in the metabolome of the samples collected from different locations. This could be because these were much less significant than the variations due to age and/or other variables such as collection time or seasonal variation. Thus, to investigate differences according to their geographical origin, same-age samples from Neimenggu, Gansu, Shanxi, and Shanxi were analysed. These four locations were chosen because the number of similar age samples was sufficient; 2–3 year-old samples and other minor samples were removed for this analysis.

**Figure 8:**
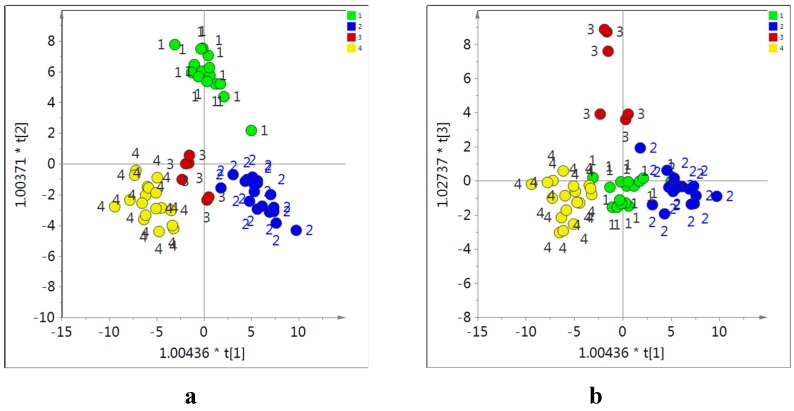
Score of orthogonal partial least square (OPLS) modeling obtained from ^1^H-NMR data of in CD_3_OD extracts of *Astragalus* roots as X-data and locations of each sample as Y-data using the combination of OPLS component 1 and 2 (**a**) and 1 and 3 (**b**). 1: Neimengu, 2: Ganxu, 3: Shanxi, 4: Shaanxi in China.

In these conditions, the four groups of Astragalus samples were well distinguished using OPLS component 1, 2 and 3 (Figure 8). The samples from Neimunggu, Shanxi and Ganxu in China were separated by the component 3, 2, and 1, respectively. The variable importance for the projection (VIP) method was used to determine which metabolites contributed to the separation, This showed that saponins, trigonelline, isoflavonoids including calycosin, formononetin and their glycosides, glucose, sucrose, citric acid, malic acid, and some amino acids (asparagine, aspartic acid and proline) were the main discriminating metabolites.

The loading plot revealed that *Astragalus* roots grown in Neimunggu had higher levels of isoflavonoids, while those from Shaanxi had more asparagine, aspartic acid, citric acid and malic acid and Gansu samples had more trigonelline and sucrose. Shanxi samples were distinguished from all others for their high content in saponins (astragaloside I, II, III, and IV) and proline. This is interesting because a recent study had concluded that the highest quality roots produced in China were from this province [10] suggesting that it could be related to the saponin content.

## 3. Experimental Section

### 3.1. Collection of Astragalus Roots and Identification

Wild or cultivated *Astragalus membranaceus* Bunge, *Astragalus membranaceus* var. mongholicus (Bunge) Hsiao or *Hedysarum polybotrys* Hand-Mazz. Roots were collected or purchased in diverse places in China during the period of 2012–2013. The detailed information of the samples employed in this study is listed in Table 2. The samples were identified by one of the authors (Lu Zheng). Voucher specimens are deposited at Shanghai Haini Pharmaceutical Co. Ltd., Shanghai, China. Each voucher specimen was prepared by triplicate.

### 3.2. Extraction and Reference Compounds

The powdered samples were extracted using a modified version of the method published by Kim* et al.* [26]. A ground sample (30 mg) was placed in a 1.5 mL-microtube and either a mixture of 500 µL of CD_3_OD and 500 µL of KH_2_PO_4_ buffer, pH 6 in D_2_O containing 0.29 mM trimethylsilyl propionic acid sodium salt-*d_4_*, (w/v, TMSP*-d_4_*, Sigma-Aldrich, St. Louis, MO, USA) or methanol-*d*_4_ (1.0 mL) containing 0.44 mM of tetramethylsilane (TMS) was added. The mixture was mixed at room temperature for 1 min, and sonicated for 5 min (Branson 5510E-MT, Branson Ultrasonics, Danbury, CT, USA). After centrifugation at 13,000 g at room temperature for 5 min, 300 µL of the supernatant were transferred to a 3 mm NMR glass tube and analyzed. The reference compounds used in this study -astragaloside I, II, III and IV, formononetin, calycosin and their 7-*O*-glucosides (>97% purity)—were purchased from Biopurify Phytochemicals Ltd. (Chengdu, China). The solutions of reference compounds were prepared in concentrations of 0.7–1.2 mg/mL of CD_3_OD or the mixture of CD_3_OD-KH_2_PO_4_ buffer in D_2_O for the NMR measurements.

### 3.3. NMR Measurements

Deuterated methanol and water were purchased from Sigma-Aldrich. ^1^H-NMR spectra were recorded at 25 °C on a 600 MHz DMX-600 spectrometer (Bruker, Karlsruhe, Germany) operating at a proton NMR frequency of 600.13 MHz and 150.13 MHz for ^13^C. CD_3_OD was used as the internal lock. The NMR experimental parameters of ^1^H-NMR, ^1^H-^1^H-correlated spectroscopy (COSY), J-resolved, heteronuclear single quantum coherence (HSQC), and heteronuclear multiple bond correlation (HMBC) followed our previous reports [27].

### 3.4. Data Analysis and Statistics

The ^1^H-NMR spectra were automatically reduced to ASCII files. Spectral intensities were scaled to total or internal standards (TMSP or TMS signals at δ 0.0) and reduced to integrated regions of equal width (δ 0.04) corresponding to the region of δ 0.0–10.0. The regions of δ 4.7–4.9 and δ 3.28–3.34 were excluded from the analysis because of the residual signal of D_2_O and CD_3_OD, respectively. Bucketing was performed by AMIX software (ver. 3.0 Bruker) with scaling on total intensity. Projections to latent structures (PLS) and orthogonal PLS (OPLS) with scaling based on Unit Variance were performed with the SIMCA-P+ software (v. 13.3, Umetrics, Umeå, Sweden).

## 4. Conclusions

The herbal medicine Radix Astragali is one of the most well-known traditional medicines in China. There are many factors involved in the quality of the plants used to produce this herbal drug. However, among them, age and location of collection in particular, are considered to be of utmost importance. So far, there have been many reports on the influence of primary metabolites in the variations of the metabolite profile but no clear connection had been established with secondary metabolites such as saponins and isoflavonoids. In this case, a NMR-based metabolomics study was done on different-age samples collected in different locations to get a more complete picture of possible metabolic variations and detect biomarkers related to these aspects and thus to quality.

On the basis of the results of the present study, two important secondary metabolite groups, saponins and isoflavonoids are proposed as chemomarkers for age and geographical location; isoflavonoids are influenced both by age and location but saponins are mostly associated to the geographical origin. As isoflavonoids are important for the therapeutical efficacy of Radix Astragali, it would be important to include its age as a requirement to guarantee its quality. This however, is mentioned in neither the Chinese nor European pharmacopeias.

The levels of the main constituents in *Astragalus* roots not only varied according to their age or location but also, probably more so, to their collection time. Ma* et al.* had reported that September to October was the best period to collect the plants because they contained the highest concentration of the main constituents such as isoflavonoids and astragaloside I as well as GABA [9]. However, it is important to note that this data is only based on up to 4-year-old roots.
